# Improved YOLOv3 Integrating SENet and Optimized GIoU Loss for Occluded Pedestrian Detection

**DOI:** 10.3390/s23229089

**Published:** 2023-11-10

**Authors:** Qiangbo Zhang, Yunxiang Liu, Yu Zhang, Ming Zong, Jianlin Zhu

**Affiliations:** School of Computer Science and Information Engineering, Shanghai Institute of Technology, Shanghai 201418, China; 216141124@mail.sit.edu.cn (Q.Z.); yuzhang@sit.edu.cn (Y.Z.); zongming@sit.edu.cn (M.Z.); 13364316620@163.com (J.Z.)

**Keywords:** occluded pedestrian detection, false positives, false negatives, loss function

## Abstract

Occluded pedestrian detection faces huge challenges. False positives and false negatives in crowd occlusion scenes will reduce the accuracy of occluded pedestrian detection. To overcome this problem, we proposed an improved you-only-look-once version 3 (YOLOv3) based on squeeze-and-excitation networks (SENet) and optimized generalized intersection over union (GIoU) loss for occluded pedestrian detection, namely YOLOv3-Occlusion (YOLOv3-Occ). The proposed network model considered incorporating squeeze-and-excitation networks (SENet) into YOLOv3, which assigned greater weights to the features of unobstructed parts of pedestrians to solve the problem of feature extraction against unsheltered parts. For the loss function, a new generalized intersection over union_intersection over groundtruth_ (GIoU_IoG_) loss was developed to ensure the areas of predicted frames of pedestrian invariant based on the GIoU loss, which tackled the problem of inaccurate positioning of pedestrians. The proposed method, YOLOv3-Occ, was validated on the CityPersons and COCO2014 datasets. Experimental results show the proposed method could obtain 1.2% MR^−2^ gains on the CityPersons dataset and 0.7% mAP@50 improvements on the COCO2014 dataset.

## 1. Introduction

Pedestrian detection is widely used in many fields to complete perception tasks [1,2,3,4]. However, there are considerable difficulties in pedestrian detection: (i) significant differences in pedestrian appearance; (ii) occlusion; and (iii) complex background. Among them, the occlusion problem has attracted much attention, and the research on occluded pedestrian detection has produced immense achievements in recent years [5,6,7,8]. However, occlusion is still regarded as an urgent problem in the field of pedestrian detection. In automatic driving scenarios, crowd occlusion is the main aspect of the occlusion problem, and the problem of crowd occlusion will lead to false positives and false negatives, which weakens the performance of pedestrian detectors. Occluded pedestrian detection methods have undergone a transition from manual feature extraction-based methods to deep learning-based methods.

Manual feature extraction-based methods feed manually extracted features into a classifier. Dala et al. proposed the histogram of oriented gradient (HOG) feature [9], which used the feature of gradient direction distribution to represent the local shape of a target, but the feature descriptor had a high dimension and the acquisition process was complicated. Dollar et al. introduced the integral channel feature (ICF) [10], using the integral map to sum up the local rectangular area of the channel image. The detection effect was outstanding when there was not much occlusion, but the adaptability to the occluded environment was poor. Felzenszwalb et al. proposed the deformable part model (DPM) [11], which built a model for the relationship between parts of a human body. Although it could solve the problem of changing pedestrian postures, the speed was slow. Although manual feature extraction-based methods have high accuracy, they need to manually extract a high dimension of features, which is time-consuming, and the detection effect on crowd occlusion scenes is poor.

Deep learning-based methods use deep neural networks to automatically extract useful features for the task, which is faster and has become the mainstream method in occluded pedestrian detection. Zhang et al. proposed a part model based on a faster region-based convolutional neural network (Faster R-CNN) [12], which combined the method of two-stage object detection with the part model, and added an attention mechanism [13,14,15] to guide the model to focus on the visible parts of the body. However, the features extracted by the convolutional part of the model were not aimed at the unobstructed part. Huang et al. proposed a post-processing method: R^2^non-maximum suppression (R^2^NMS) [16], which used the visible part of the pedestrian to reduce false positives, but it caused the problem of false negatives. Ref. [17] proposed a novel NMS algorithm to refine the predicted boxes. However, the algorithm generated a small number of false negatives in the crowd scenes. Ref. [18] proposed a model to estimate a set of highly overlapped pedestrians for each proposal, which could be applied to numerous proposal-based detectors. However, false negatives were generated when encountering extremely crowded scenes. Ref. [19] applied the model of the transformer to crowd detection, which focused on the unobstructed parts of pedestrians. However, the positioning accuracy of a pedestrian was poor. Chi et al. proposed a novel joint head and human detection network to detect the head and human body simultaneously in the crowd scenes [20], but there were false negatives in the heavily overlapped scenes. Although deep learning-based methods are faster, the efficiency of classification and the degree of the positioning accuracy of occluded pedestrians are low and the number of false negatives is high.

To address the problem of false positives and false negatives caused by crowd occlusion, an improved you-only-look-once version 3 (YOLOv3) based on squeeze-and-excitation networks (SENet) and optimized generalized intersection over union (GIoU) loss, called YOLOv3-Occlusion (YOLOv3-Occ), is proposed. It uses uncovered parts to accurately classify and locate pedestrians in crowd occlusion scenes. The contributions of the proposed YOLOv3-Occ are summarized as follows:

- The channel attention mechanism of squeeze-and-excitation networks (SENet) is adopted to be incorporated between the feature extraction layers of YOLOv3, which gives larger weights to the features of the non-overlapping parts of pedestrians to address the problem of feature extraction of uncovered parts.
- The positional loss function of Generalized Intersection over Union_Intersection over Groundtruth_ (GIoU_IoG_) is proposed by replacing IoU in the GIoU loss function with IoG, which makes the areas of predicted frames of pedestrians constant to solve the issue of inaccurate location of pedestrians.

The rest of this paper is arranged as follows: Section 2 introduces the related works, including loss function works and network model works. Section 3 presents the proposed YOLOv3-Occ method, including preliminary works, the architecture of YOLOv3-Occ, and the optimized loss function. Section 4 describes the experimental results and analysis. Section 5 concludes this paper.

## 2. Related Works

### 2.1. Loss Function Works for Pedestrian Location

The accuracy of pedestrian localization can be improved by optimizing the positional loss function. Mean square error loss (MSE Loss) [21] calculated the mean value of the square of the Euclidean distance between the predicted tensor and the target tensor in n-dimensional space, but the loss value changed drastically in the early stage of training and was sensitive to outliers. Wang et al. [22] proposed smooth_L1_ loss, which used the l_1_ and l_2_ norms of the distance vector between the predicted tensor and the target tensor. However, this loss function was not equivalent to IoU and did not take the relevance of the coordinates of bounding boxes into account. The IoU positioning loss function [23] was proposed by Yu et al., where the coordinates of bounding boxes were regarded as a whole to construct the loss function, but when the prediction frame and the target frame were disjoint, the loss function could not be optimized. The GIoU loss function [24] was proposed by R et al., which introduced the normalized area between the prediction box and the target box. But when the prediction frame and the target frame intersected, the loss function could be optimized by reducing the area of the prediction frame, causing the prediction frame to be far away from the target frame. Ref. [25] proposed the measurement standard of IoG, changing the denominator of IoU to the area of the target box to make the prediction box close to the target box. Ref. [26] proposed two repulsion losses, which introduced penalties to predictions that overlapped with considerable ground truths and predictions. However, the weights of the two losses were not evaluated by experiments. Ref. [27] proposed NMS loss, which added the penalty of false positives and false negatives to the loss to reduce them in the training process. However, it was only suitable for binary classification tasks. Previous loss function works did not solve the problem that a prediction area reduces when optimizing a loss function. To overcome the problem, GIoU_IoG_ loss is proposed in the paper. The loss function works are summarized in Table 1.

### 2.2. Network Model Works for Occluded Pedestrian Detection

It is imperative to design a robust network model to handle crowd occlusion. Occlusion-aware region-convolutional neural network (OR-CNN) [28] used part-based models, which divided pedestrians into several parts and merged the results of the part detection as the final result. Since every part was represented as a rectangle, the model would produce noise. Ref. [29] proposed a set of decision trees capturing the overall distribution of all parts, which were shared by the part detectors. A channel attention network was proposed to add to the CNN method [30], using channel-wise attention to focus on the unobstructed parts of the occludee. Ref. [31] proposed an attention-guided neural network model (AGNN), which selected features representing the body parts of pedestrians. Previous network model works did not transfer visible part features to detection branches at different scales in the mission of multi-scale object detection. To solve this drawback, SENet is incorporated into YOLOv3 in the proposed method. The network model works are summarized in Table 2.

## 3. Proposed Method: YOLOv3-Occ

In this part, YOLOv3-Occ is proposed to address the problem of crowd occlusion. SENet is used to be integrated into the feature extraction layer of the YOLOv3 network model, giving the unobstructed parts larger attention, which solves the problem of key body parts being occluded. GIoU_IoG_ loss is proposed as the positioning loss function, making the prediction frame quickly approach the target frame, which solves the problem of the prediction frame rejecting the target frame. Soft-NMS is adopted as the post-processing method. In summary, SENet makes the category and bounding box parameters more accurate, and then reduces the initial value of GIoU_IoG_ loss. GIoU_IoG_ loss makes the positioning of the model more accurate, which in turn makes the workload of Soft-NMS smaller and improves the reasoning speed. The three modules work together to reduce false negatives and false positives in the scene of crowd occlusion.

### 3.1. Preliminary Work

#### 3.1.1. SENet

**Figure 1:**
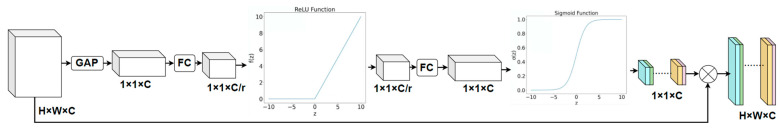
The architecture of SENet. The input is a feature map. The output is the feature map injected with the attention scores. Firstly, the GAP layer compresses the shape of the input feature map to (1, 1, C). Secondly, two fully connected layers and activated functions obtain the attention scores of all channels, as shown in the feature map marked by diverse colors. Finally, the operation of multiplying channel by channel is implemented between the input feature map and the attention scores to generate the output.

SENet is a channel attention network, which introduces attention scores in the channel dimension. The architecture of SENet is shown in Figure 1, which is mainly divided into three modules: (1) Global Average Pooling (GAP) layer for compressing the shape of the input feature map to (1, 1, C); (2) Multi-Layer Perception (MLP) for obtaining the attention scores of all channels, as shown in the feature map marked by diverse colors in Figure 1; and (3) scale operation to obtain the original feature map injected with the attention scores. The ReLU activation function in MLP is shown in Formula (1):

c_i_^ReLU^ = max(0, c_i_^1^), (1)

where max is the maximum function, c_i_^1^ is one of values of the nodes output by the first fully connected layer, and c_i_^ReLU^ is c_i_^1^ activated by the ReLU function.

#### 3.1.2. GIoU Loss

GIoU loss is a positioning loss function based on IoU loss, which introduces the smallest enclosing rectangle to optimize the situation where the prediction and the target do not overlap. GIoU loss is shown in Formula (2):

GIoU loss = 1 − [IoU − (S^S^ − S^U^)/S^S^], (2)

where S^U^ is the union area of the prediction box and the target box, and S^S^ is the area of the smallest external rectangle.

#### 3.1.3. Soft-NMS

Soft-NMS is a post-processing method based on NMS, which introduces a one-dimensional Gaussian kernel function to reduce the confidences of significantly overlapped predictions rather than discarding the predictions. The kernel function is shown in Formula (3):

k(IoU(M, d_i_)) = e^−IoU(M, di)*IoU(M, di)/σ^, (3)

where M is the prediction frame with the highest confidence at present, d_i_ is one of the remaining prediction frames after removing M, IoU(M, d_i_) is the IoU between M and d_i_, and σ is the hyperparameter which needs to be adjusted. Equation (3) is the Gaussian kernel function with a mean of 0 and a variance of σ.

### 3.2. The Architecture of the Proposed YOLOv3-Occ

**Figure 2:**
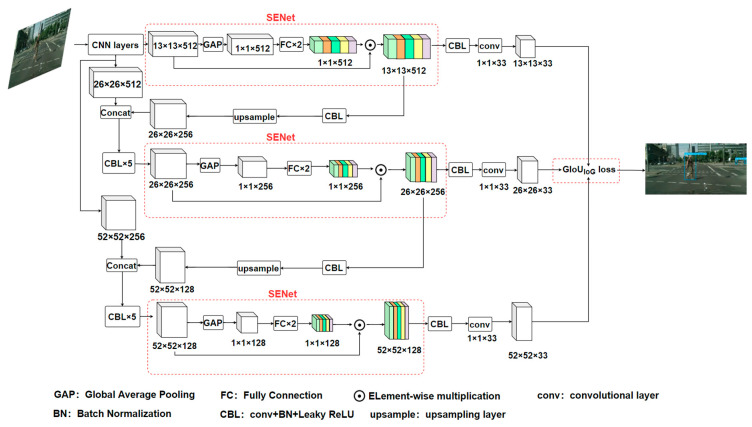
The architecture of the proposed YOLOv3-Occ. For an input image, the output is the image with detection boxes. The black arrow represents the data flow. CNN layers consist of a set of CBLs and basic residual blocks, which are used to extract fine-grained features of the input image. The outputs of two basic residual blocks in CNN layers are used as the inputs of two Concat layers in the model.

Figure 2 shows the architecture of YOLOv3-Occ. For an input image, the output is the image with detection boxes. Note that three SENets [32] are incorporated into the prediction branch of three scales, respectively, so that the feature channels of the visible parts on pedestrians can be given larger weight. In addition, the outputs of the first two SENets are fused with the outputs of two basic residual blocks in CNN layers, respectively, so the features of the visible part in the previous scale branch are used as the input of the SENet in the subsequent scale branch to refine the features. Therefore, the accuracy of pedestrians’ classification and positioning in the three scales could be improved by harnessing the refined features of the unobstructed parts. The proposed GIoU_IoG_ loss is used to compute the positioning loss and optimize the model. The specific information about the loss is described in Section 3.3.

### 3.3. The Proposed Loss Function: GIoU_IoG_ Loss

GIoU_IoG_ loss is the positioning loss function of YOLOv3-Occ. It is proposed by replacing IoU in GIoU loss with IoG. The computation process of GIoU_IoG_ loss is shown in Algorithm 1. The input is the coordinates of the upper left corner and the lower right corner of the prediction box and the target box, and the output is the function value of GIoU_IoG_ loss. The time complexity of Algorithm 1 is O(1) since there are no circulated and recursive processes, and the space complexity is O(1) since the required memory of the algorithm does not change with the problem size. GIoU_IoG_ loss is shown in Formula (4):

GIoU_IoG_ loss = 1 − [IoG − (S^S^ − S^U^)/S^S^], (4)

where S^U^ is the union area of the prediction box and the target box, and S^S^ is the area of the smallest external rectangle. When there is no intersection between the prediction box and the target box, IoG is equal to 0. While optimizing (4) in the situation, the vertices of the prediction frame are moved to the direction where the prediction frame and the target frame overlap. When there is an intersection between the prediction frame and the target frame, IoG is greater than 0. While optimizing (4) in this case, the intersection area between the prediction box and the target box increased instead of reducing the prediction area like GIoU loss. Therefore, the accuracy of localization can be improved by harnessing the loss function whether in the overlapped scenes or non-overlapped scenes.
**Algorithm 1:** The computation process of GIoU_IoG_ loss**input:** the coordinates of the upper left corner and the lower right corner of       prediction(B^p^) and ground truth(B^g^):      B^p^ = (x_1_^p^, y_1_^p^, x_2_^p^, y_2_^p^), B^g^ = (x_1_^g^, y_1_^g^, x_2_^g^, y_2_^g^) **output:** GIoU_IoG_ loss **Step 1** Calculating the coordinates of the common area(I) of B^p^ and B^g^:      x_1_^I^ = maximum(x_1_^p^, x_1_^g^), y_1_^I^ = maximum(y_1_^p^, y_1_^g^), x_2_^I^ = minimum(x_2_^p^, x_2_^g^),       y_2_^I^ = minimum(y_2_^p^, y_2_^g^)      where (x_1_^I^, y_1_^I^) is the coordinates of the upper left corner, and (x_2_^I^, y_2_^I^) is the       coordinates of the lower right corner. **Step 2** Calculating the area of I:      S^I^ = (x_2_^I^ − x_1_^I^) × (y_2_^I^ − y_1_^I^)      where x_2_^I^ − x_1_^I^ is the width of I, and y_2_^I^ − y_1_^I^ is the height of I. **Step 3** Calculating the area of B^p^:      S^p^ = (x_2_^p^ − x_1_^p^) × (y_2_^p^ − y_1_^p^)      where x_2_^p^ − x_1_^p^ is the width of B^p^, and y_2_^p^ − y_1_^p^ is the height of B^p^. **Step 4** Calculating the area of B^g^:      S^g^ = (x_2_^g^ − x_1_^g^) × (y_2_^g^ − y_1_^g^)      where x_2_^g^ − x_1_^g^ is the width of B^g^, and y_2_^g^ − y_1_^g^ is the height of B^g^. **Step 5** Calculating the area of the union between B^p^ and B^g^:      S^U^ = S^p^ + S^g^ − S^I^      where the reason for the operation, minus S^I^, is that S^I^ is calculated twice in the       calculation process of S^p^ + S^g^. **Step 6** Calculating the IoG:      IoG = S^I^/S^g^      where IoG is the ratio of the intersection area to the target area. **Step 7** Calculating the coordinates of the smallest external rectangle(B^s^) surrounding B^p^
      and B^g^:      x_1_^S^ = minimum(x_1_^p^, x_1_^g^), y_1_^S^ = minimum(y_1_^p^, y_1_^g^), x_2_^S^ = maximum(x_2_^p^, x_2_^g^),       y_2_^S^ = maximum(y_2_^p^, y_2_^g^)      where (x_1_^S^, y_1_^S^) is the coordinates of the upper left corner, and (x_2_^S^, y_2_^S^) is the       coordinates of the lower right corner. **Step 8** Calculating the area of B^s^:      S^s^ = (x_2_^S^ − x_1_^S^) × (y_2_^S^ − y_1_^S^)      where x_2_^S^ − x_1_^S^ is the width of B^s^, and y_2_^S^ − y_1_^S^ is the height of B^s^. **Step 9** Calculating the GIoU_IoG_:      GIoU_IoG_ = IoG − (S^s^ − S^U^)/S^s^      where GIoU_IoG_ is generated by replacing IoU in GIoU with IoG. **Step 10**  Calculating the GIoU_IoG_ loss:      GIoU_IoG_ loss = 1 − GIoU_IoG_

## 4. Experimental Results and Analyses

### 4.1. Experiment Settings

#### 4.1.1. Datasets

An ideal method of pedestrian detection for crowd occlusion scenes should be robust to instance distributions, i.e., not only effective for crowded detections but also stable for detecting a single person. Two datasets, CityPersons [33] and COCO2014 [34], are adopted for comprehensive evaluations on moderately and slightly occluded scenes, respectively. Table 3 lists the sizes and overlaps of the datasets. Table 4 shows different annotation types for the datasets. The categories of CityPersons are divided into six classes: fake humans, pedestrians, riders, sitting persons, other persons with unusual postures, and groups of people; and COCO2014 uses 80 classes: person, bicycle, car, and other common categories in life. The size of an image in CityPersons is 1024 × 2048 pixels and in COCO2014 480 × 640 pixels. Since the proposed approach aims to improve the performance of crowded detections, numerous experiments are performed on CityPersons. In addition, experiments on COCO2014 are performed to verify whether the proposed method undermines uncrowded detections.

#### 4.1.2. Evaluation Metrics

Precision (P), Recall (R), Average Precision_50_ (AP_50_), mean AP@50 (mAP@50), and log-average Miss Rate on False Positive Per Image (FPPI) in [10^−2^, 100] (MR^−2^) are used as the evaluation metrics of the model:

- Both P and R are for a single category of a single picture. Larger P and R indicate better performance. The formula of P and R are shown in (5) and (6), respectively:
  P = (true positives)/(true positives + false positives),(5)
  R = (true positives)/(true positives + false negatives),(6)
- AP_50_ is aimed at a single category of all pictures, which is the area enclosed by the P–R curve and the R axis when the iou-threshold is 0.5. It is used to measure the performance of the model in a given category. The larger the AP_50_ is, the better the performance is.
- mAP@50 is the mAP when the iou-threshold is 0.5, which is used to measure the performance of the model in all categories. The larger the mAP@50 is, the better the performance is. The formula of mAP@50 is shown in (7):
  mAP@50 = (1/C)Σ_c=1_^C^AP_50_^c^, (7)
  where c is one category, C is the number of classes, and AP_50_^c^ is the AP_50_ of the class represented by c.
- MR^−2^, the area enclosed by the MR-FPPI curve and the FPPI axis, is commonly used in pedestrian detection. A smaller MR^−2^ suggests better performance.

#### 4.1.3. Detailed Settings

For the anchor setting, the same scale and shape [35] are used. Mini-batch Gradient Descent (MGD) is used as the optimized algorithm. We set the batch size as 32 images since the batch size could reach the fastest training speed under the GPU memory. As is seen in Table 5, the initial learning rate is set to 10^−3^, which is used to train the first 65 epochs, and the learning rate is reduced by 10 times in the last 20 epochs, set to 10^−4^. At the same time, the gradual warmup is used in the first 1000 steps of training. As the number of steps increases, the learning rate increases slowly. When the training step reaches the 1001st step, the constant learning rate is used for training.

### 4.2. Experiments on CityPersons

All the pedestrian detectors are trained on the training set of CityPersons and evaluated on the validation set. The settings of the training and validation process are shown in Table 5.

#### 4.2.1. Ablation Study

Table 6 shows the ablation experiments of the proposed method in Section 3, including the SENet module and GIoU_IoG_ loss. It is explicit that the performances in all criteria are basically improved by adding two contributions, respectively. Specifically, adding the GIoU_IoG_ loss causes P to obtain a 1.1% improvement which is the peak of two improvements in P, indicating that the loss could improve the accuracy of the match between prediction frames and target frames as we expected. In addition, integrating the SENet module gives a 0.8% improvement to R which is the maximum of two improvements in R, suggesting that more true positives could be found. More importantly, adding two contributions simultaneously boosts all the evaluation metrics, indicating that all contributions are compatible with each other.

#### 4.2.2. Comparisons with Previous Works

Table 7 lists some other state-of-the-art methods on the CityPersons validation set. Although our approach reduces the mAP@50 by 45.6% over the method of EMD-RCNN, the MR^−2^ of it transcends most of the listed methods. Especially, it boosts the MR^−2^ by 1.2% and 0.8% over the method of Adaptive-NMS and MGAN, respectively. SENet is incorporated into the network model to make the class and positional parameters more accurate and GIoU_IoG_ loss is proposed to make the positioning of the prediction more accurate. The two modules help reduce the number of false positives. Soft-NMS is used to retain predictions of other targets, which reduces the number of false negatives. Finally, the MR^−2^ of the model is reduced.

#### 4.2.3. The Impact of the Hyperparameters on YOLOv3-Occ

**Figure 3:**
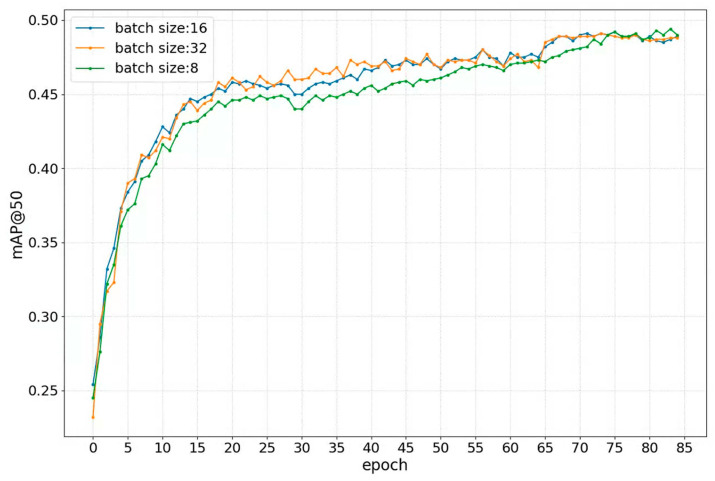
Comparison of mAP@50s among three batch sizes on the CityPersons training set. The curves show how the mAP@50s of these batch sizes change with the number of epochs. The three batch sizes and their corresponding curve colors are represented in the upper left corner of the figure.

Figure 3 shows the change in mAP@50 under different batch sizes. All curves present a similar trend, rising rapidly and then remaining stable. Specifically, when the batch size is set to 32, mAP@50 reaches the peak the fastest and spends 20 epochs reaching the peak. However, mAP@50 achieves the summit the slowest against the batch size of eight. It indicates that the batch size influences the training speed of the proposed method and the larger batch size under the GPU memory produces the faster training speed.

**Figure 4:**
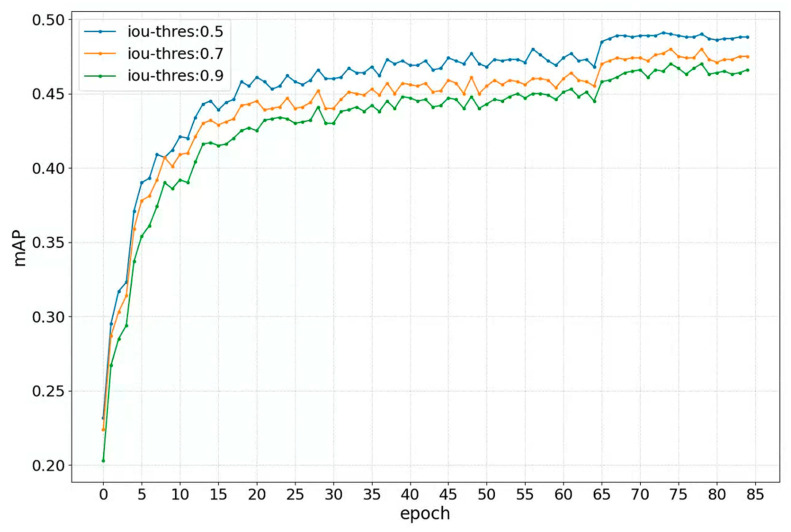
Comparison of mAPs among three iou-thresholds on the CityPersons training set. The curves show how the mAPs of these iou-thresholds change with the number of epochs. The three iou-thresholds and their corresponding curve colors are represented in the upper left corner of the figure.

Figure 4 shows the change in mAP under diverse iou-thresholds. The iou-threshold is used to judge whether a prediction is a true positive. All curves reach the peaks at almost the same speed. Note that the peak of mAP is the biggest under the iou-threshold of 0.5 and is the smallest under the iou-threshold of 0.9. It suggests that the mAP performance of the proposed method is affected by the iou-threshold and the mAP performance is better with the smaller iou-threshold.

#### 4.2.4. Visual Comparison

**Figure 5:**
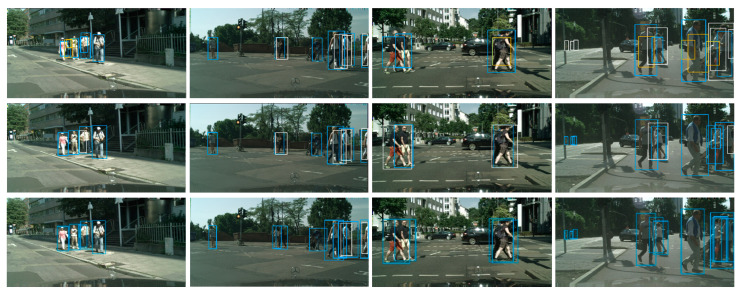
Visual comparison of the baseline, EMD-RCNN, and our approach. The first row is the results of the baseline. The second row is the results generated by EMD-RCNN. The third row is the results of YOLOv3-Occ. The blue boxes are the detection results, the white boxes are false negatives, and the yellow boxes are false positives.

The visualized results of our method on the CityPersons validation dataset are shown in Figure 5. The visualization threshold is set to 0.5 to remove the redundant boxes in the results. For example, the third column is the outputs of the image through the three models. It can be seen in the last output that the proposed method could detect the four pedestrians accurately without false positives and false negatives. However, there is one false positive and two false negatives in the output of the baseline model and EMD R-CNN, respectively. Therefore, the proposed method could reduce the number of false positives and false negatives in the crowd occlusion scenes as we expected.

### 4.3. Experiments on COCO2014

According to Table 3, the crowdedness of the COCO2014 dataset is relatively low, which is not our design purpose. Therefore, a significant performance gain on the dataset is not expected. The reason why the dataset is introduced is to validate whether the proposed method is robust to different crowdedness levels. All the object detectors are trained on the training set of COCO2014 and evaluated on the validation set. For a fair comparison, most of the involved methods are retrained under the same settings in Table 5.

#### 4.3.1. Ablation Study

Table 8 shows the ablation experiments of the proposed method on the COCO2014 dataset. It can be seen in the second and third rows that the performances in all indexes are promoted by adding two contributions, respectively. It is noted that SENet also plays a more important role in the increase in R and GIoU_IoG_ loss with the increase in P. In addition, adding the two contributions simultaneously improves all the evaluation metrics. Therefore, the SENet and the GIoU_IoG_ loss not only work on the CityPersons dataset but also on the COCO2014 dataset.

#### 4.3.2. Robustness Experiments

**Figure 6:**
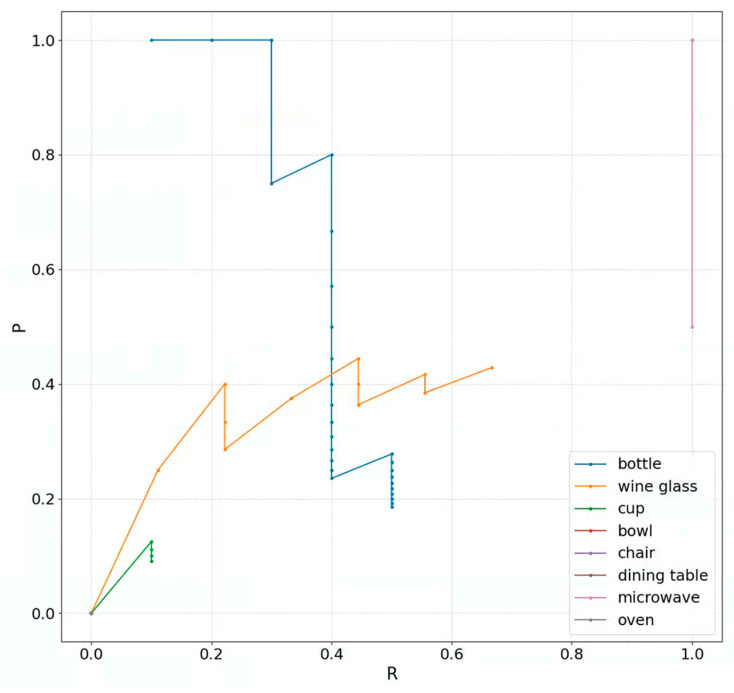
P–R curve on one batch of the COCO validation set. There are eight classes of P–R curves and four of them coincide with the rest of the curves. The eight classes and their corresponding curve colors are represented in the lower right corner of the figure.

**Figure 7:**
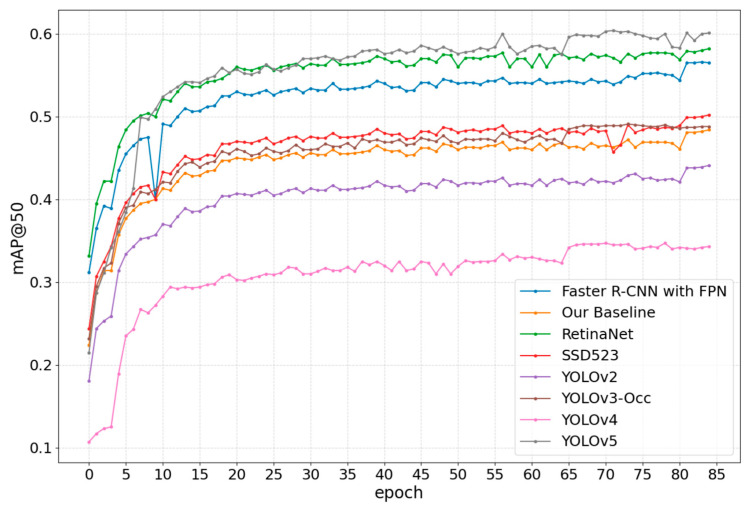
Comparison of mAP@50s among YOLOv3-Occ, Faster R-CNN with FPN [38], our baseline, RetinaNet [39], SSD523 [40], YOLOv2, YOLOv4 [41], YOLOv5 [42] on the COCO validation set. The curves show how the mAP@50s of these methods change with the number of epochs. The eight methods and their corresponding curve colors are represented in the lower right corner of the figure.

Figure 6 shows the P–R curve plotted based on the validated results of the proposed method. The areas enclosed by many curves and the R-axis are greater than 0, indicating that the AP_50_s of the classes represented by these curves are relatively high. Figure 7 compares the mAP@50 of other methods and YOLOv3-Occ. All curves exhibit similar trends, with mAP@50 plateauing after around the 20th epoch. However, compared to the baseline, YOLOv2, and YOLOv4, the performance of YOLOv3-Occ continues to be better, and finally, it is about 0.7%, 4.7%, and 15.4% higher than the three methods, respectively. The experiments suggest our method is also able to deal with relatively uncrowded scenes without a significant drop in performance.

### 4.4. Computation Cost and Limitation

Compared with YOLOv3, a limitation of the proposed method is that it is time-consuming. Table 9 shows the time-related indexes of the two methods. After adding the proposed contributions to YOLOv3, the parameter quantity and average training time per epoch slightly increased. The incorporation of SENet produces the increase in the parameter quantity as shown in column 2 of Table 9, while the integration of SENet and the improvement in GIoU loss causes the rise of the training time as shown in column 3 of Table 9. Although the proposed method improves MR^−2^, the training cost of the method rises slightly. Although the training time of the proposed method is longer than for YOLOv3, the inference time of the proposed method is almost the same as for YOLOv3.

## 5. Conclusions and Future Directions

This paper proposes a novel method of occluded pedestrian detection in crowd scenes: YOLOv3-Occ. Based on YOLOv3, SENet is adopted to be integrated into the feature extraction layer, and GIoU_IoG_ loss is proposed as the positioning loss function. The ablation experimental results on the CityPersons dataset show that P, R, and mAP@50 have been improved by 2.0%, 1.8%, and 2.4%, respectively, after adding the two contributions. Experimental results show that the MR^−2^ of YOLOv3-Occ is relatively high compared with a series of the state-of-the-art methods, which reaches an advanced level. Meanwhile, experiments are performed on the COCO2014 dataset to test the generality of the two contributions and validate the robustness of YOLOv3-Occ.

In summary, YOLOv3-Occ reduces the false positives and false negatives of pedestrians under the scenes of crowd occlusion and is robust to numerous degrees of occlusion. However, YOLOv3-Occ faces new challenges, especially regarding poor performance in scenes of severe pedestrian occlusion and multi-class object detection. Therefore, the next step is to analyze the reasons for the poor performance. According to these reasons, we need to find out solutions, such as integrating suitable attention mechanisms into suitable positions in the network model, developing a new loss function, and improving the post-processing method.

## Figures and Tables

**Table 1:** The loss function works for pedestrian locations.

Achievements	Effect	Disadvantage
MSE Loss [21]	Euclidean distance between a prediction and a target	Drastic change in the loss
Smooth_L1_ Loss [22]	The l_1_ and l_2_ norms of the distance vector between a prediction and a target	Inequivalent to IoU
IoU Loss [23]	Coordinates of bounding boxes regarded as a whole	Unoptimizable when a prediction and a target are disjoint
GIoU loss [24]	The normalized area between a prediction and a target supplementing the IoU loss	Changing areas of prediction frames during the optimization of the loss
Repulsion Loss [26]	Loss of predictions overlapped with other ground truths and predictions	The unevaluated weights of two losses
NMS Loss [27]	The penalty of false positives and false negatives supplementing the loss	Only suitable for binary classification tasks

**Table 2:** The network model works for occluded pedestrian detection.

Achievements	Effect	Disadvantage
OR-CNN [28]	Divide pedestrians into several parts	Noise production
Multi-label Learning [29]	A set of decision trees shared by the part detectors	/
Guided Attention [30]	Channel-wise attention to pay attention to the unobstructed parts of the occludee	/
AGNN [31]	Select features representing the body parts of pedestrians	/

**Table 3:** Volume and overlapped extent of each dataset. The overlap of an image is the average of the overlaps of all people in the image. The overlap of a person = 1 − (the area of the visible box)/(the area of the full box).

Dataset	Size of Training Set/Imgs	Size of Validation Set/Imgs	Size of Test Set/Imgs	Overlaps per Img
CityPersons	2975	500	1575	0.32
COCO2014	117,264	5000	—	0.015

**Table 4:** Annotation types of each dataset. Full bbox denotes the box of the full body of a pedestrian, visible bbox the box of visible parts of a pedestrian, and head bbox the box of a pedestrian’s head; Bbox: Bounding box. The symbol of ✓ denotes that the box exists in the annotation of the dataset and the symbol of 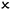 denotes the non-existence of the box.

Dataset	Full Bbox	Visible Bbox	Head Bbox
CityPersons	✓	✓	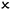
COCO2014	✓	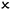	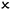

**Table 5:** Parameter settings: σ is the variance of the Gaussian kernel function used in the Soft-NMS; iou-threshold is the standard to judge as a true positive.

Name of Parameters	Value of Parameters
device	NVIDIA GeForce RTX 3090 from USA
GPU memory	24 GB
batch size	32
epoch	85
learning rate	10^−3^ (epoch ≤ 65); 10^−4^ (epoch > 65)
momentum	0.9
σ	0.5
iou-threshold	0.5

**Table 6:** Ablation experiments evaluated on the CityPersons validation set. The baseline model (the first line) is YOLOv3. SE—SENet. GL—GIoU_IoG_ Loss.

SE	GL	P/%	R/%	mAP@50/%
		49.7	50.6	48.1
✓		50.5	51.4	49.6
	✓	50.8	51.2	49.4
✓	✓	51.7	52.4	50.5

**Table 7:** Comparisons of different methods on the CityPersons validation set.

Method	Backbone	MR^−2^/%	mAP@50/%
EMD-RCNN [18]	ResNet-50	10.7	96.1
NMS-Ped [27]	ResNet-50	10.1	—
CSP [36]	ResNet-50	11.0	—
Adaptive-NMS [17]	VGG-16	11.9	—
MGAN [37]	VGG-16	11.5	—
Ours	Darknet-53	10.7	50.5

**Table 8:** Ablation experiments evaluated on the COCO2014 validation set. The baseline model is YOLOv3 (the first line). SE—SENet. GL—GIoU_IoG_ Loss.

SE	GL	P/%	R/%	mAP@50/%
		48.3	49.6	47.5
✓		48.8	50.3	48.1
	✓	49.3	50.1	48.6
✓	✓	50.5	51.9	49.7

**Table 9:** Comparison of time-related indexes in two methods on the CityPersons training and validation set. YOLOv3 is the original method without adding the proposed contributions. YOLOv3-Occ is the proposed method. M: million. S: seconds. FPS: Frame Per Second. #: The Number of.

Method	# Parameters/M	Average Training Time per Epoch/S	FPS in the Inference Process/Imgs
YOLOv3	61	1.33	3
YOLOv3-Occ	62	2.40	3

## Data Availability

Data are contained within the article.
